# Integrated PPI- and WGCNA-Retrieval of Hub Gene Signatures Shared Between Barrett's Esophagus and Esophageal Adenocarcinoma

**DOI:** 10.3389/fphar.2020.00881

**Published:** 2020-07-31

**Authors:** Asma Sindhoo Nangraj, Gurudeeban Selvaraj, Satyavani Kaliamurthi, Aman Chandra Kaushik, William C. Cho, Dong Qing Wei

**Affiliations:** ^1^ The State Key Laboratory of Microbial Metabolism, Department of Bioinformatics and Biostatistics, School of Life Science and Biotechnology, Shanghai Jiao Tong University, Shanghai, China; ^2^ Center of Interdisciplinary Sciences-Computational Life Sciences, Henan University of Technology, Zhengzhou, China; ^3^ Wuxi School of Medicine, Jiangnan University, Wuxi, China; ^4^ Department of Clinical Oncology, Queen Elizabeth Hospital, Hong Kong, China; ^5^ Peng Cheng Laboratory, Shenzhen, China

**Keywords:** bioinformatics analysis, Barrett's esophagus, hub gene signature, esophageal adenocarcinoma, weighted gene co-expression network analysis, protein–protein interaction

## Abstract

Esophageal adenocarcinoma (EAC) is a deadly cancer with high mortality rate, especially in economically advanced countries, while Barrett's esophagus (BE) is reported to be a precursor that strongly increases the risk of EAC. Due to the complexity of these diseases, their molecular mechanisms have not been revealed clearly. This study aims to explore the gene signatures shared between BE and EAC based on integrated network analysis. We obtained EAC- and BE-associated microarray datasets GSE26886, GSE1420, GSE37200, and GSE37203 from the Gene Expression Omnibus and ArrayExpress using systematic meta-analysis. These data were accompanied by clinical data and RNAseq data from The Cancer Genome Atlas (TCGA). Weighted gene co-expression network analysis (WGCNA) and differentially expressed gene (DEG) analysis were conducted to explore the relationship between gene sets and clinical traits as well as to discover the key relationships behind the co-expression modules. A differentially expressed gene-based protein–protein interaction (PPI) complex was used to extract hub genes through Cytoscape plugins. As a result, 403 DEGs were excavated, comprising 236 upregulated and 167 downregulated genes, which are involved in the cell cycle and replication pathways. Forty key genes were identified using modules of MCODE, CytoHubba, and CytoNCA with different algorithms. A dark-gray module with 207 genes was identified which having a high correlation with phenotype (gender) in the WGCNA. Furthermore, five shared hub gene signatures (SHGS), namely, pre-mRNA processing factor 4 (PRPF4), serine and arginine-rich splicing factor 1 (SRSF1), heterogeneous nuclear ribonucleoprotein M (HNRNPM), DExH-Box Helicase 9 (DHX9), and origin recognition complex subunit 2 (ORC2), were identified between BE and EAC. SHGS enrichment denotes that RNA metabolism and splicosomes play a key role in esophageal cancer development and progress. We conclude that the PPI complex and WGCNA co-expression network highlight the importance of phenotypic identifying hub gene signatures for BE and EAC.

## Introduction

Esophageal cancer is a deadly cancer considering its high mortality rate, with 572,034 newly diagnosed cases and 508,585 deaths in 2018 (Bray et al., 2018). Esophageal cancer is classified into two subcategories: esophageal adenocarcinoma (EAC; distal esophagus) and esophageal squamous cell carcinoma (ESCC; proximal esophagus). It starts from the esophageal epithelium, the innermost layer of the esophagus (Rustgi and El-Serag, 2014). Esophageal cancer is a very complex disease, as its various subtypes have different risk factors, time trends, and geographic patterns (Analysis et al., 2017) (Montgomery et al., 2014; Lordick et al., 2016). According to the geographic variation, EAC is more common in economically advanced regions than in low-income countries (Chai et al., 2019). The common risk factors of EAC are age, male sex, obese, gastroesophageal reflux disease (GERD), cigarette smoking, and diet (low in vegetables and fruits). Cook et al. (2014) report some common symptoms like vomiting/nausea and heartburn in EAC and GERD. Besides, Barrett's esophagus (BE) is considered as a precursor for EAC. BE is a metaplastic transformation from the normal squamous mucosa of the esophagus to a columnar lining; its presence conveys a 30–40-fold increased risk of EAC (Schneider and Corley, 2015). The tumor development is a step-by-step process that comprises constant changes from erosive esophagitis to non-dysplastic BE, low-grade dysplasia, high-grade dysplasia, adenocarcinoma *in situ*, and finally invasive adenocarcinoma (Anaparthy and Sharma, 2014). Due to poor prognosis, over 40% of patients are diagnosed with high-grade dysplasia. Additionally, the 5-year survival rate is less than 20% despite the advances in diagnosis and treatment (Tramontano et al., 2017). Certainly, surgical therapy has improved the patient's survival yet it is not suitable for advanced-stage cancer patients (Davies et al., 2014).

Thus, it is essential to discover biomarkers that can lead to the discovery of medication. Microarray analysis of gene expression profiles is a common practice for identifying key hub genes and key pathways (Wei et al., 2018; Sadhu and Bhattacharyya, 2019). In the current era of integrated bioinformatics, acquiring data is not an issue; rather, normalization seems to be a tough job (Campain and Yang, 2010). Considering all of these notions, we designed an integrated study to find key hub genes associated with BE and EAC. First, we extracted BE- and EAC-associated microarray datasets from the Gene Expression Omnibus (GEO) and ArrayExpress using systematic meta-analysis as well as RNA-seq data from TCGA. Preprocessing and normalization were conducted for further analysis. DEGs were identified using linear models for microarray data (LIMMA) algorithm. Meta-analysis was performed using a network analysis tool. We analyzed functional and pathway enrichment of DEGs. Additionally, a protein–protein interaction (PPI) network was constructed to study the associations between the DEGs and to recognize target genes using different modules of Cytoscape software. Weighted gene co-expression network analysis (WGCNA) was conducted by the construction of the co-expression network to find a correlation between modules and clinical traits. Furthermore, clinically significant modules were identified. Finally, key hub genes were identified and validated using immunohistochemistry and survival analysis.

## Materials and Methods

### Data acquisition, Preprocessing, and Normalization

**Figure 1:**
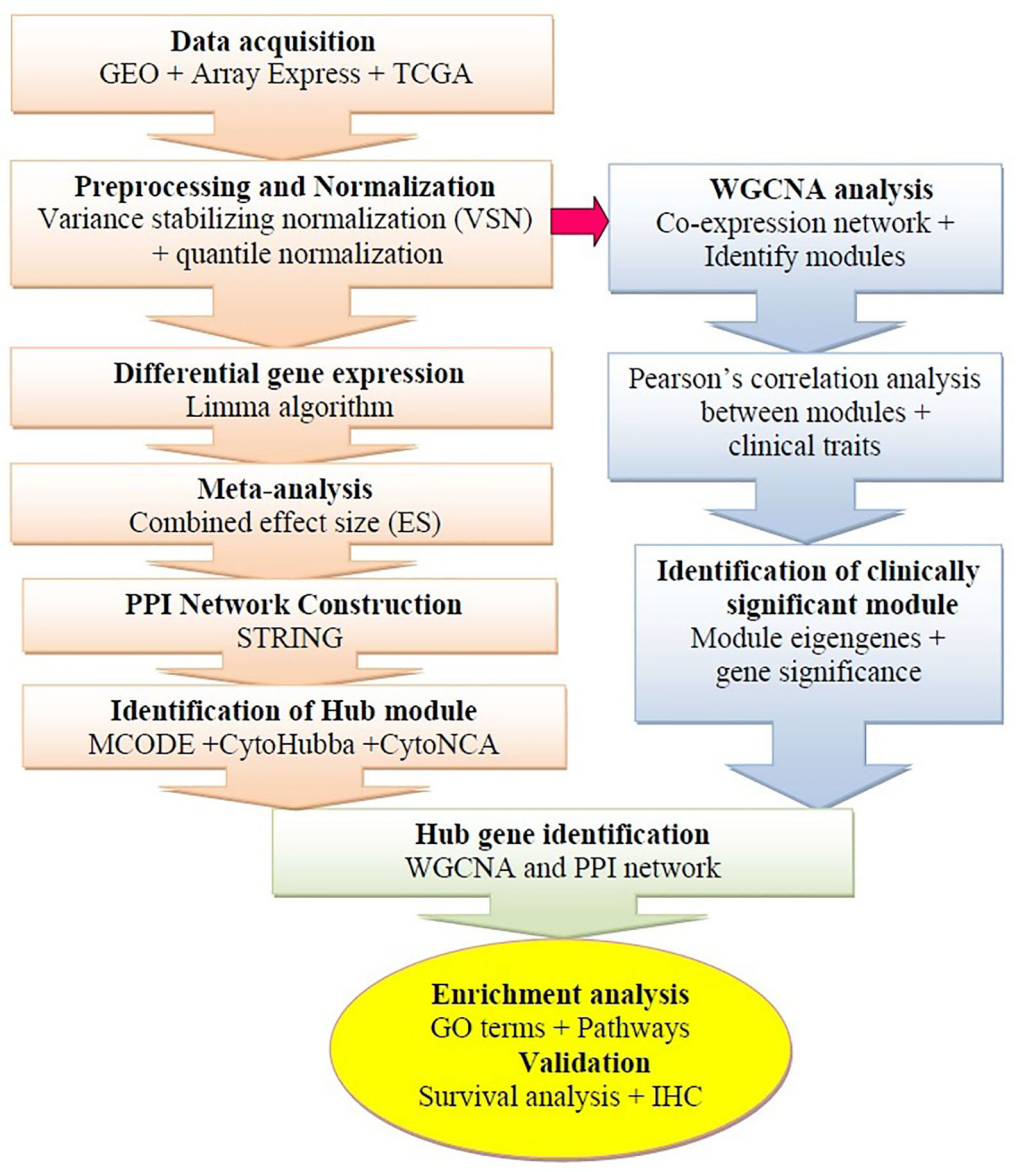
Schematic flow diagram of the study. GEO, Gene Expression Omnibus; IHC, Immunohistochemistry; WGCNA, weighted gene co-expression network analysis; TCGA, The Cancer Genome Atlas; PPI, Protein-protein interaction.

The microarray datasets were systematically extracted from the GEO^1^ (Edgar et al., 2002) and the ArrayExpress^2^ database (Brazma et al., 2003). The gene expression profiles based on RNA-sequencing were additionally obtained from The Cancer Genome Atlas (TCGA)^3^ (Zhu et al., 2014). The framework of this study is shown in **Figure 1**. For microarray profiles, we selected four datasets (GSE26886, GSE1420, GSE37200, and GSE37201) available by October 2019 (Kimchi et al., 2005; Silvers et al., 2010; Wang et al., 2013; Lin et al., 2015). The GEO accession number, sample size, description, platform, expression data, and references are extracted from each identified dataset (**Table 1**). The TCGA portal was accessed in October 2019, 184 esophageal cancer samples were retrieved. The tab-delimited text (.txt) files of microarray datasets were obtained from the GEO database. The Network Analyst (NA) web interface for integrative biological network analysis was employed for background correction preprocessing, normalization, probe identification, and meta-analysis of the datasets (Xia et al., 2015). The input files were prepared as per the description of the tool (first line #Name (sample ID); second line #class (sample type); genes in the rows and samples in the columns). We applied two different methods to normalize the datasets: first, variance stabilizing normalization (VSN), which improves DEG detection and reduces false-positive errors (Konishi, 1985), and second, quantile normalization, which can make two distributions equal in statistical methods (Hansen et al., 2012). The processed datasets were used for subsequent microarray meta-analysis.

**Table 1:** Relevant information about selected microarray datasets.

GSE Acc. No.	No. of Samples	Platform	Description	Country	PMID
GSE26886	21 vs 20	AHG-U133 Plus 2.0 Array	Gene expression profiling of Barrett's esophagus, adenocarcinoma, esophageal squamous epithelium, and squamous cell carcinoma	Germany	23514407
GSE1420	8 vs 8	AHG-U133A Array	Barrett's esophagus, Barrett's-associated adenocarcinomas and normal esophageal epithelium	USA	15833844
GSE37200	15 vs 31	AHG-U133A Array	Gene expression profiling of Barrett's esophageal tissues and esophageal adenocarcinoma	USA	26068949
GSE37201	22	AHG-U133A Array	Barrett's esophageal tissues and esophageal adenocarcinoma	USA	20332323

AHG, Affymetrix Human Genome.

### DEG Identification and Meta-Analysis

Differential gene expression analysis was performed with the R package LIMMA (linear models for microarray data), which is embedded in NA (Ritchie et al., 2015). Each gene expression was calculated based on the false discovery rate (FDR; *p* < 0.05) using the Benjamini–Hochberg method and *t*-test. In addition, the microarray meta-analysis between EAC and BE samples was performed using combined effect size (ES). The combined ES is the difference between two group means divided by standard deviation, which is comparable across different studies. It can be calculated by two types of models, namely fixed-effect models (FEM) and random-effect models (REM). In FEM, the calculated effect size in each study is supposed to arise from an original true effect size plus measurement error. In REM, each study further contains a random effect that can incorporate unknown cross-study (different platforms) heterogeneities in the model. The FEM or REM can be chosen based on statistical heterogeneity estimated using Cochran's Q tests (Cochran, 1950). The method typically gives a lower number of DEGs but more confidence (Selvaraj et al., 2018).

Cochran's Q test equation:

Cochran’s Q test equation: T=k(k-1)∑j=1k(X·j−NK)2∑i−1bXi·(k−Xi·)

where *k* is the number of samples; *X · _j_* is the column total for the *j* ^th^ sample; *b* is the number of genes; *X_i_ ·* is the row total for the *i* ^th^ gene; *N* is the grand total.

### Gene Ontology and Pathway Enrichment Analysis

We used ClueGO v2.5.3, a Cytoscape^4^ plugin, for function and pathway enrichment analysis of DEGs (Bindea et al., 2009; Kohl et al., 2011). A list of DEGs or hub genes were provided as input into ClueGO with select specific parameters, for example, species, such as *Homo sapiens,* ID type—Entrez gene ID, different enrichment functions—biological process or cellular component or molecular function or KEGG pathways, for the analysis. Each enrichment was calculated based on the Bonferroni method (kappa score 0.96; cutoff value *p* < 0.005).

### PPI Network Construction and Module Extraction

The search tool for retrieval of interacting genes/proteins (STRING)^5^ (Szklarczyk et al., 2017) is a database that is used to construct the PPI network. Currently, the database consists of 18,838 human proteins with 25,914,693 core network interactions. In this study, we constructed the PPI network from identified DEGs using the STRING interactome. The highest confidence interaction score was set to 0.9, which reduces the number of false-positive interactions (Bozhilova et al., 2019). Molecular complex detection (MCODE) is a Cytoscape plugin used to identify the finest clusters. MCODE calculates accurate correlation levels as well as identifying essential PPI network modules (Shannon et al., 2003). In addition, other add-ins of Cytoscape, namely, CytoHubba and CytoNCA, were employed to discover the highest linkage hub genes in the network (Chen et al., 2009; Tang et al., 2015).

### WGCNA Analysis

The WGCNA package was employed to construct a gene co-expression network using a variant set of genes (12,701 genes). The analysis was performed based on the package instructions (Langfelder and Horvath, 2008). The connection strength between each pair of nodes was calculated using the adjacency matrix *a_ij_*.

$$Z_{ij}=\left[cor\left(b_{i},b_{j}\right)\right]a_{ij}=Z_{ij\beta }$$

While vectors (*bi* and *bj*) were expression values for genes, Pearson's correlation coefficient of gene *i* and *j* and *a_ij_* were represented as the connection strength between genes. The soft-thresholding power of *β* = 9 was used to ensure scale-free topology. The hierarchical clustering of the weighting coefficient matrix was used to define the modules. The functional modules in the co-expression network with defined genes, the topological measure (TOM) indicating the concurrence in shared adjacent genes, was calculated as

$$TOMi,j=\frac{\Sigma^{N}{}_{K\;=1}A_{i,j}.\;A_{k,j}+\;A_{i,j}}{min\left(K_{i,}K_{j}\right)\;+\;1-\;A_{i,j}}$$

where *A* is the weighted adjacency matrix described in the above formula. TOM-based dissimilarity measures with a minimum size of 100 for the gene dendrogram and average linkage hierarchical clustering were performed, and similar expression profiles were divided into the same gene modules using the dynamic tree cut package.

### Identification of Clinically Significant Modules

Eigengene and gene significance methods were used to identify modules that were correlated with clinical traits of the GSE37200 microarray data set. The first principal component of each gene module and the expression of the module eigengene were defined as representative of the whole gene set and were described in the first eigengene module. The association between module eigengenes and clinical trait was used to calculate and identify the significant clinical module. Second, the gene significance was described as a mediated *p-*value of each gene in the linear regression between expression and clinical traits. Furthermore, the module significance was described as the average the gene significance of all genes associated with the module. The average absolute gene significance was defined as module significance. It was calculated to incorporate clinical traits into a co-expression network (Langfelder and Horvath, 2008).

### Survival Analysis and Validation of SHGS

The SHGS were identified from the modules of WGCNA and the PPI network using an interactive Venn diagram. The R package *survival* was employed to calculate Kaplan–Meier (KM) survival plots with hazard ratio (HR) and log-rank tests of hubs, which was implemented in the *OSeac* ^6^ (**consensus survival analysis for EAC) web interface. *OSeac* retrieved the gene expression profiles and clinical data including TNM (Stage I, II, III, and IV), gender (male and female), race (White, Black, and African American), and grade (G1, G2, G3, and GX) of 198 patients from TCGA and GEO. We analyzed the overall survival rate of the shared gene signature as an input and obtained the plot from the tool (Wang et al., 2020). The Human Protein Atlas^7^ was used to validate the immunohistochemistry of SHGS (Uhlén et al., 2005; Uhlen et al., 2017).

## Results

### Physiognomies of Selected Studies

We collected a total of 682 studies from the GEO and ArrayExpress database up to October 2019. In all, 678 datasets/studies that did not satisfy the inclusion criteria were excluded. Finally, four potential studies were selected (**Supplementary Figure 1**). Among the four selected studies, three were conducted on the Affymetrix human genome U133A platform and one was performed on the Affymetrix human genome U133 plus 2.0 platform, which included 125 samples in total chosen in this study. In each study, EAC samples were compared with the adjacent BE samples. The dataset GSE37200 was used to construct a co-expression network with the relevant clinical trait information. After preprocessing and normalization, the GSE37200 dataset with 22,284 genes was further processed, and variant genes (12,701) were selected for WGCNA studies.

### Identification of DEGs and Enrichment Analysis

**Figure 2:**
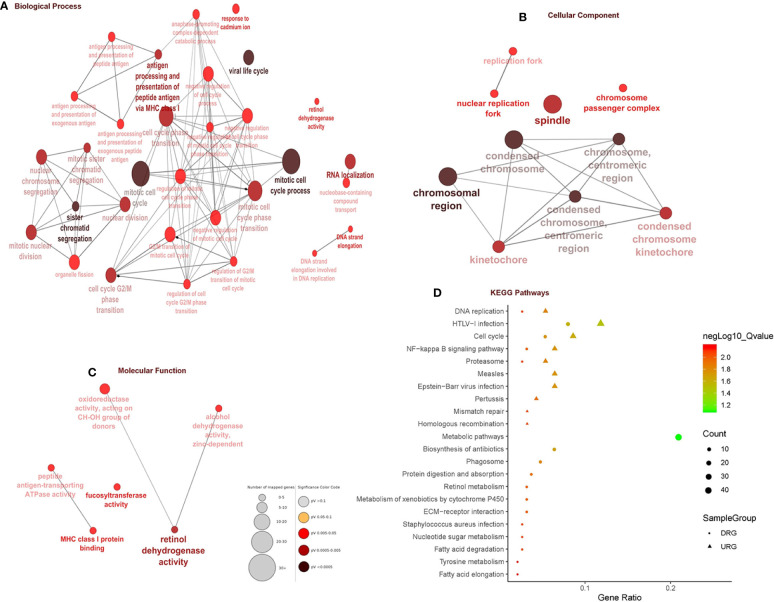
Gene ontology and pathway enrichment analysis. **(A)** Biological process analysis. **(B)** Cellular component analysis. **(C)** Molecular function analysis. **(D)** KEGG pathway analysis.

In total, 403 DEGs were obtained through microarray meta-analysis, which include 169 downregulated and 234 upregulated genes. A heatmap is a simple yet effective way to compare the content of multiple major gene lists. Major DEGs across all the datasets were represented in red, orange, and yellow in a heatmap. Gray indicates that the respective gene is not present in the gene list (**Supplementary Figure 2**). **Table 2** illustrates the top 10 upregulated and downregulated DEGs. Monocyte differentiation antigen CD14 (CD14), ribose 5-phosphate isomerase A (RPIA), tumor necrosis factor superfamily member 11 (TNFSF11), plexin D1 (PLXND1), major histo-compatibility complex, class II DM beta (HLA-DMB), and spliceosome-associated factor 3, and U4/U6 recycling protein (SART3) were highly expressed upregulated genes, whereas fucosyltransferase 2 (FUT2), SECIS binding protein 2 like (SECISBP2L), COP9 signalosome subunit 4 (COPS4), gelsolin (GSN), and glutathione peroxidase 3 (GPX3) were highly expressed downregulated genes. According to the gene ontology (GO) terms BP, MF, and CC, downregulated genes were significantly enriched in the mitotic cell cycle process, sister chromatid segregation, antigen processing, presentation of peptide antigen *via* MHC class I, chromosomal region, and MHC class I protein binding, whereas retinol dehydrogenase activity and fucosyltransferase activity were highly enriched in upregulated genes associated with EAC (**Figures 2A–C**). In KEGG, pathway enrichment demonstrated that the upregulated genes were enriched for viral myocarditis, cell cycle, DNA replication, and AGE-RAGE signaling pathways in diabetic complications. Downregulated genes were associated with pathways involved in fatty acid degradation, glycosphingolipid biosynthesis, and amino sugar and nucleotide sugar metabolism (**Figure 2D**).

**Table 2:** Top ten up- and downregulated genes.

S.No.	Gene	Gene name	Combined ES	P-value
**Upregulated genes**		
1	CD14	Monocyte differentiation antigen CD14	1.2948	4.98E-08
2	RPIA	Ribose 5-phosphate isomerase A	1.1376	4.63E-08
3	TNFSF11	Tumor necrosis factor super family member 11	1.1229	3.66E-08
4	PLXND1	Plexin D1	1.1223	3.60E-08
5	HLA-DMB	Major histo-compatibility complex, class II, DM beta	1.1168	3.97E-08
6	SART3	Spliceosome associated factor 3, U4/U6 recycling protein	1.1142	4.06E-08
7	OSBPL3	Oxysterol binding protein like 3	1.1119	4.79E-08
8	PRAF2	PRA1 domain family member 2	1.1117	4.61E-08
9	PILRB	Paired immunoglobin like type 2 receptor beta	1.1078	4.67E-08
10	RGS16	Regulator of G protein signaling 16	1.1064	4.79E-08
**Downregulated genes**		
1	FUT2	Fucosyltransferase 2	-1.1095	4.67E-08
2	SECISBP2L	SECIS binding protein 2 like	-1.111	4.52E-08
3	COPS4	COP9 signalosome subunit 4	-1.1148	4.79E-08
4	GSN	Gelsolin	-1.117	4.04E-08
5	GPX3	Glutathione peroxidase 3	-1.1185	3.87E-08
6	ADH1A	Alcohol dehydrogenase 1A (class I), alpha polypeptide	-1.1271	4.39E-08
7	CORO2A	Coronin 2A	-1.2074	4.67E-08
8	ACADS	Acyl-CoA dehydrogenase short chain	-1.2157	3.57E-08
9	RCAN2	Regulator of calcineurin 2	-1.2172	5.07E-08
10	CLEC3B	C-type lectin domain family 3 member B	-1.2316	3.73E-08

*Combined ES, Cochran's combination test of random effect model (REM) or effect size (ES).

### WGCNA and Clinically Significant Module Identification

**Figure 3:**
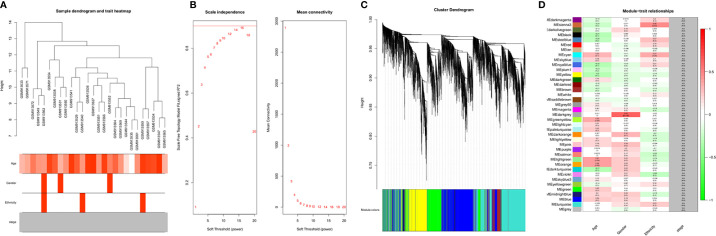
**(A)** Clustering dendrogram of samples with trait heatmap. **(B)** Analysis of network topology for various soft-thresholding powers. The left panel shows the scale-free fit index (y-axis) as a function of the soft-thresholding power (x-axis). The right panel displays the mean connectivity (degree, y-axis) as a function of the soft-thresholding power (x-axis). **(C)** Clustering dendrogram of genes, with dissimilarity based on topological overlap, together with assigned module colors. **(D)** Module-trait associations: Each row corresponds to a module eigengene and each column to a trait. Each cell contains the corresponding correlation and p-value. The table is color-coded by correlation according to the color legend. Distribution of average gene significance and errors in the modules associated with gender and ethnicity of cancer patient.

A dendrogram of samples (GSE37200) with clinical trait was clustered using the average linkage method and Pearson's correlation method (**Figure 3A**). Co-expression analysis was carried out to construct the co-expression network. In this study, the power of *β* = 9 (scale-free *R* ^2^ = 0.95) was selected as the soft-thresholding parameter to ensure a scale-free network (**Figure 3B**). A dendrogram of all differentially expressed genes was clustered based on a dissimilarity measure (1-TOM) (**Supplementary Figure 3**). A total of 39 modules were identified through hierarchical clustering. Light green (eigengene value = 0.41), dark gray (eigengene value = 0.62) and Sienna3 (eigengene value = 0.46) modules appeared to have the highest association with age, gender, and ethnicity. There was no module–trait relationship associated with tumor stage, denoted as NA in **Figure 3C**. Therefore, the dark gray module having the highest association with gender was selected as the clinically significant module for further analysis. There were 207 phenotypic genes identified in the dark-gray module (**Figure 3D**). In **Supplementary Figure 4**, the hierarchical clustering dendrogram of the eigengene network represents the relationships among the modules and the clinical trait weight.

### Identification and Validation of Hub Genes

**Figure 4:**
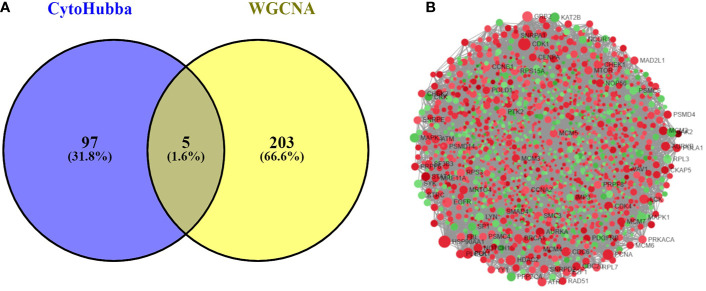
**(A)** Venn diagram demonstrates overlapping genes of the DEG-PPI network and WGCNA. **(B)** DEG-PPI network complex (upregulated genes showed in green; downregulated genes showed in red).

**Figure 5:**
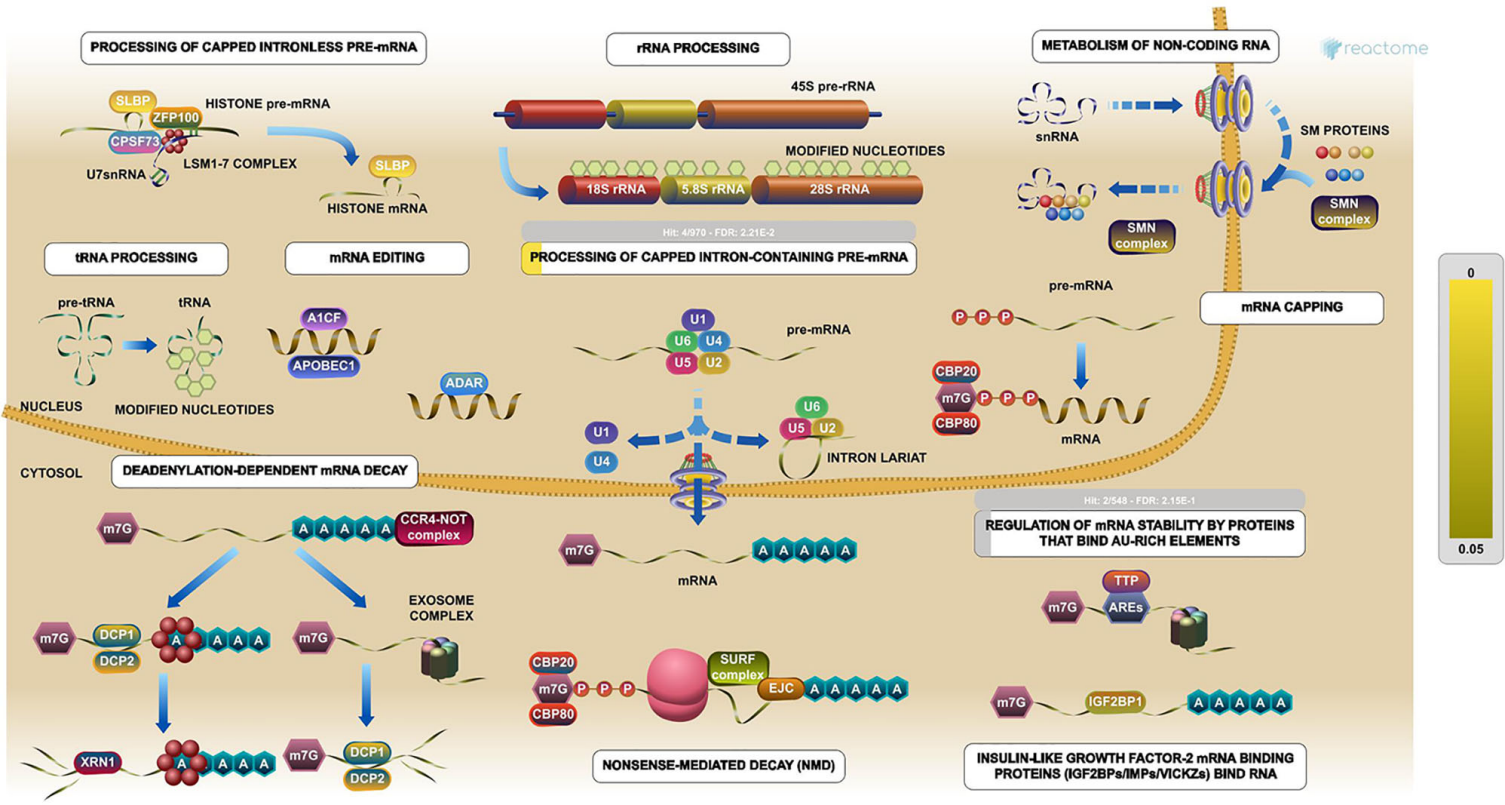
Pathway enrichments of SHGS. PRPF4, SRSF1, HNRNPM, and DHX9 are keys genes in the RNA metabolic pathway. These genes especially are involved in the preprocessing of capped intron-containing pre-mRNA and regulation of mRNA stability by proteins that bind AU-rich elements. (Image extracted from the Reactome pathway analyzer).

The PPI network was constructed with 403 DEGs using the STRING database. The interactive relationships between the key genes in the whole network were determined using the Cytoscape plugins (MCODE, Cytoscape, and CytoHubba). There are two clusters: 82 nodes and 938 edges in cluster 1, and 20 nodes and 168 edges in cluster 2, which were identified from MCODE based on a scoring system (cutoff k-score = 12). In addition, the data were imported into another plugin, CytoHubba, which helped to identify 104 key genes through five different calculation methods, namely, EPC, MCC, DMNC, MNC, and Stress. Then, the two clusters were imported into the CytoNCA plugin, which helped to identify 40 key genes using five different algorithms, namely, betweeness, closeness, degree, eigenvector, and subgraph. We securely conceive that the key genes are the intersections between the PPI network and the dark-gray module with 207 genes (**Supplementary Table 1**) highly correlated with phenotype (gender) from the WGCNA analysis (**Figures 4A, B**). Finally, five SHGS, namely, pre-mRNA processing factor 4 (PRPF4), serine and arginine rich splicing factor 1 (SRSF1), heterogeneous nuclear ribonucleoprotein M (HNRNPM), DExH-box helicase 9 (DHX9), and origin recognition complex subunit 2 (ORC2), are identified between BE and EAC. Pathway enrichment demonstrated that all the SHGS are involved in the metabolism of RNA, and its molecular functional terms include cell cycle, DNA binding, DNA topoisomerase binding, pre-mRNA splicing, and RNA helicase activity (**Figure 5**).

### Survival Analysis and Immunohistochemistry

**Figure 6:**
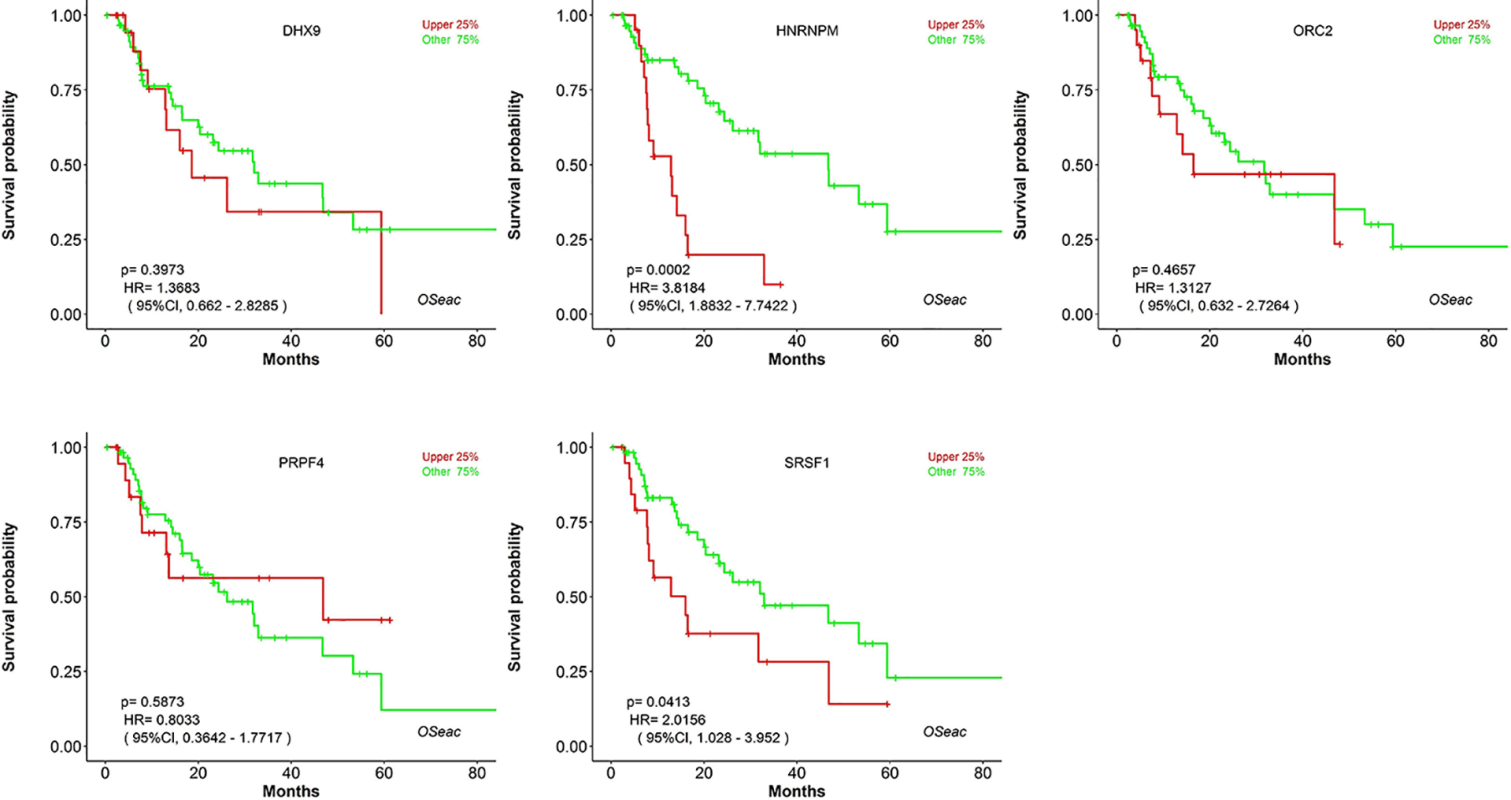
The prognostic value of hub genes in BE and EAC patients (Kaplan–Meier plot).

**Figure 7:**
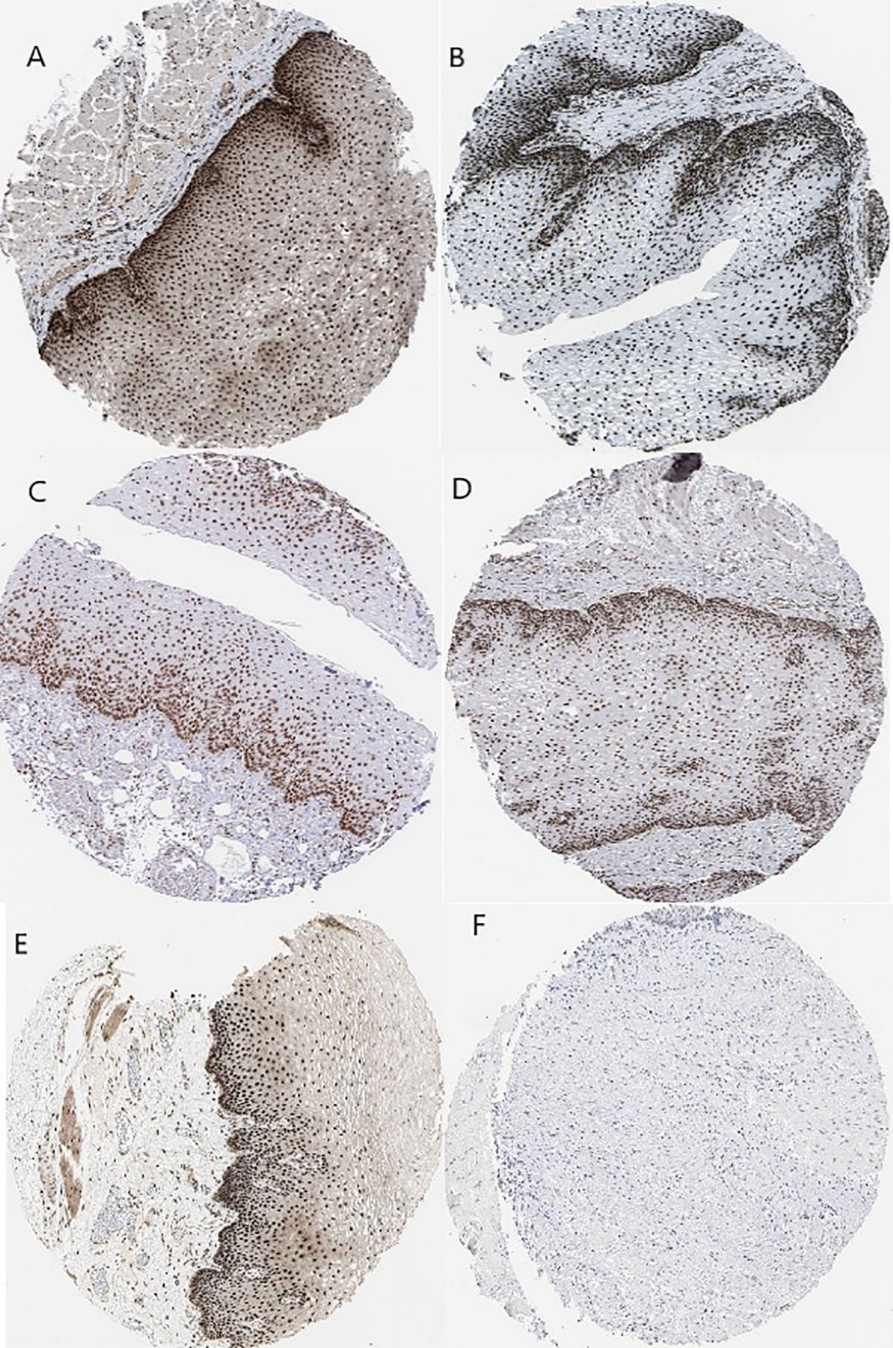
Immunohistochemistry of the five hub genes based on the Human Protein Atlas. **(A)** Protein levels of PRPF4 in normal tissue (staining: high; intensity: strong; quantity: >75%). **(B)** Protein levels of SRSF1 in normal tissue (staining: high; intensity: strong; quantity: >75%). **(C)** Protein levels of SRSF1 in tumor tissue (staining: high; intensity: strong; quantity: >75%). **(D)** Protein levels of HNRNPM in normal tissue (staining: high; intensity: strong; quantity: >75%). **(E)** Protein levels of DHX9 in normal tissue (staining: high; intensity: strong; quantity: >75%). **(F)** Protein levels of ORC2 in normal tissue (staining: not detected; intensity: low; quantity: <25%).

Kaplan–Meier plots demonstrated that the prognostic impact of the SHGS was identified from modules of the PPI network complex and WGCNA. The results revealed that high expression of HNRNPM and SRSF1 was associated with poor overall survival of BE and EAC patients (*p* < 0.05). Moreover, high expression of PRPF4, DHX9, and ORC2 was correlated with longer overall survival of BE and EAC patients (**Figure 6**). In addition, we plotted a gender-based survival curve to determine the correlation of WGCNA modules. The hazard ratio (HR) and 95% confidence interval were as follows in males: PRPF4 (HR =1.08; 95%CI – 0.46 ± 2.48; *p* = 0.865); SRSF1 (HR =3.08; 95%CI – 1.49 ± 6.37; *p* = 0.002); HNRNPM (HR =3.295; 95%CI – 1.54 ± 7.02; *p* = 0.002); DHX9 (HR =1.39; 95%CI – 0.64 ± 2.48; *p* = 0.404); ORC2 (HR =1.25; 95%CI – 0.58 ± 2.72; *p* = 0.564). Further, in female cases PRPF4 (HR =0.39; 95%CI – 0.03 ± 3.89; *p* = 0.421); SRSF1 (HR =1.49; 95%CI – 0.20 ± 10.79; *p* = 0.689); HNRNPM (HR =8.06; 95%CI – 0.82 ± 79.01; *p* = 0.073); DHX9 (HR =0.38; 95%CI – 0.04 ± 3.89; *p* = 0.424); ORC2 (HR =3.24; 95%CI – 0.20 ± 51.91; *p* = 0.4061). The results clearly demonstrated that the high expression of SHGS correlated to the poor prognosis of male compared to female. Furthermore, immunohistochemical slides of the Human Protein Atlas database indicated that the protein expressions of SHGS were drastically higher in cancerous tissues compared with in adjacent normal tissues, as shown in **Figure 7**. Therefore, these SHGS were all key genes that play an initiative role and might have a tendency to co-express.

## Discussion

EAC is an obstinate type of cancer, which has a high mortality rate because of poor prognosis, metastatic rate, and treatment resistance (Tatarian and Palazzo, 2019). EAC usually arises from a premalignant variation in the lining of the esophagus known as BE (Thrift, 2016). Unfortunately, the treatment and diagnosis of EAC and BE are limited due to the lack of precise molecular targets. Therefore, we designed this study to explore SHGS between EAC and BE to improve the diagnosis and prognosis status of the patients. There are numerous advanced technologies that can quantify the enormous amount of transcripts in a parallel manner. Microarray and data mining are well-known approaches for cancer biomarker discovery (Selvaraj et al., 2019). Nevertheless, a single microarray dataset is not enough to deal with this obstinate disease. However, a comprehensive analysis of a number of microarray datasets with different platforms can assist with identifying the molecular mechanism of EAC and BE. Therefore, we selected four different microarray datasets to identify SHGS and the associated pathways between BE and EAC. Moreover, WGCNA is a powerful tool for searching effective biological mechanisms and key genes from gene expression microarrays. It provides module construction and correlation analysis within the gene expression data to determine the associations between genes. It also elucidates the biological significance of a gene module to provide insights into molecular and pathological characteristics in many diseases. All these characteristics make it a robust, reliable, and significant method for analysis of large-scale data. There is no prior research employing WGCNA to do gene co-expression network analysis with BE and EAC. To explore SHGS, we decided to construct a gene co-expression network with relevant clinical trait information from the GSE37200 dataset.

Phenotype variants like age, gender, and ethnicity are factors that are intensively involved in the prognosis and diagnosis of BE and EAC (Ford et al., 2005; Runge et al., 2015). EAC usually appears at the later stage of life, but it may start at a young age in the form of BE. Earlier studies have reported that male patients with BE are at low risk of malignant progression and predominantly die due to causes other than EAC (Sikkema et al., 2010). There are studies reported that there is a marked male prevalence of EAC with a male-to-female ratio of 9:1 due to sex hormone factors. Androgen exposure may increase the risk of EAC compared to estrogen (Xie and Lagergren, 2016; Kim et al., 2016). Furthermore, geographically White people, especially White Americans, are at higher risk than other ethnicities (Schneider and Corley, 2015). A comprehensive study from January 2006 to December 2017 reported that high risk of male patients with esophageal diseases in the province of Madinah in Saudi Arabia is due to a variety of factors, including inflammatory disorders, infection, and neoplastic condition (Albasri et al., 2019). In addition, genomic analysis by restriction fragment length polymorphism indicated that the highest frequencies of Y-chromosomal haplogroups are associated with BE and EAC in White males (Westra et al., 2020). Recent case–control studies demonstrate that gastroesophageal reflux disease in male patients is highly associated with the development of BE in Germany (Schmidt et al., 2020). These reports supported the present results, indicating that predicted dark gray modules with the highest association with gender must have a clinically significant module.

Two types of biological materials, namely, GO and KEGG pathway data, are key to understanding the disease mechanism. CD14 acts as a co-receptor with toll-like receptors (TLRs) to identify evading pathogens and improve the immune system. It is reported that TLRs 1-10 are expressed in the normal esophagus and that there is a high association of TLRs 4, 5, and 9 with BE and EAC (Kauppila and Selander, 2014). TNFSF11 is a key regulator of interactions between T cells and dendritic cells, which regulate the T-cell-dependent immune response and enhance bone-resorption in hypercalcemia of malignancy (Luan et al., 2012). Somja et al. (2013) observed that both metaplastic and malignant lesions of the esophagus are infiltrated by regulatory T cells. They concluded that soluble factors secreted by epithelial cells during the EAC or BE influence tumor progression through tolerogenic dendritic cells, which can be a potential therapeutic tool. In addition, different cohort studies have reported that GSN is a serum glycoprotein biomarker used as a diagnostic tool for EAC and BE (Shah et al., 2015; Shah et al., 2018). Glycosphingolipid biosynthesis is an important pathway that can produce cell-surface glycans. These glycans are altered in the development from BE into EAC, with specific changes in lectin binding patterns. This binding is a key marker in endoscopic visualization of high-grade dysplastic lesions (Bird-Lieberman et al., 2012). These reports suggest that the predicted GO terms and pathways of DEGs are highly associated with EAC and BE.

We have identified five different SHGS (PRPF4, SRSF1, HNRNPM, DHX9, and ORC2) between EAC and BE. PRPF4, SRSF1, and HNRNPM are U4/U6 small nuclear ribonucleoprotein Prp4, serine and arginine-rich splicing factor 1, and heterogeneous nuclear ribonucleoprotein M coding genes, respectively. These genes play an important role in pre-mRNA splicing and spliceosome assembly (Bertram et al., 2017). Pre-mRNA splicing is key to the pathology and has a substantial role in generating multiple oncogenic and tumor-suppressor proteins after the post-transcriptional process. Splicing is of different types such as amino acid addition, exon skipping, frame shift, intron retention, promoter usage, truncated C-terminus, and 5′-SS, which have various clinical applications including proliferation, metastasis, drug resistance, and radiotherapy (Guo et al., 2015; Di et al., 2019). In addition, there are studies reporting splicing signatures associated with the prognosis of esophageal cancer (Lin et al., 2018; Mao et al., 2019). Through the splicing mechanism, PRPF4, SRSF1, and HNRNPM regulate the cell proliferation, migration, and invasion in different cancers, including lung cancer (Choi, 2012; Chang and Lin, 2019), breast cancer (Anczuków et al., 2015; Sun et al., 2017; Park et al., 2019), cutaneous squamous cell carcinoma (Zhang et al., 2018), hepatocellular carcinoma (Tu et al., 2019), esophagus dysplasia (Varghese et al., 2015; Fitzgerald et al., 2018), gastric cancer (Wu et al., 2019), cervical cancer (Dong et al., 2019), and Ewing's sarcoma (Passacantilli et al., 2017).

DHX9 is an ATP-dependent RNA helicase A coding gene involved in DNA replication, transcriptional activation, post-transcriptional RNA regulation, mRNA translation, and RNA-mediated gene silencing (Capitanio et al., 2017). Knockdown of ATP-dependent RNA helicase inhibited the expression of β-catenin, c-Myc, and cyclin D1 in esophageal cancer cells through suppressing the Wnt/β-catenin signaling pathway (Ma et al., 2017). In addition, ATP-dependent RNA helicase was reported to dysregulate distinct steps of mRNA and pre-ribosomal RNA metabolism in cancer cells (Awasthi et al., 2018). ORC2 is an origin recognition complex subunit 2 coding gene binding origins of replication (Shen et al., 2012). It can bind to different histone trimethylation proteins and stabilize leucine-rich repeat and WD repeat-containing protein 1 (LRWD1) through protecting it from ubiquitin-mediated proteasomal degradation (Chan and Zhang, 2012). Studies demonstrated that increased expressions of certain histone-mediated proteins correlate with advanced TNM stages, tumor grade, metastatic potential, and decreased overall and disease-free survival of patients with esophageal cancer (Schizas et al., 2018). This supportive information enhances the understanding of why the predicted DHX9, HNRNPM, ORC2, PRPF4, and SRSF1 genes are highly correlated to EAC and BE progression and act as potential biomarkers for diagnosis as well as prognosis.

## Conclusion

This network pharmacology-based study provides new insights into BE and EAC patients for their diagnosis and prognosis. The results of microarray dataset-based PPI networks and WGCNA exhibited that the dark-gray module had the maximum association with EAC and BE, with the identification of five SHGS, namely PRPF4, SRSF1, HNRNPM, DHX9, and ORC2. The WGCNA-based gene co-expression network indicated that the relationships between co-expressed genes and clinical trait (gender of the patient) were associated with the progression of esophageal cancer. SHGS enrichment denotes that the RNA metabolic and spliceosome pathways play an essential role in the development and progress of esophageal cancer. Survival analysis demonstrates that the high expression of HNRNPM and SRSF1 in esophageal cancer might be a poor prognostic marker. The co-expression modules were established to preserve a reliable expression relationship independent of phenotype and may share similar biological functions. This approach shares the limitations of other data mining methods: the results of WGCNA can technically be biased due to tissue contamination or artifacts. To enhance the reliability of the WGCNA results, immuno-histochemical data from the Human Protein Atlas were used for confirmation. However, we could not obtain all the related IHC data of tumor and adjacent normal samples for each gene due to the database constraint. These findings may support new therapeutic targets and potential useful for the advancement of prognostic biomarker evaluation.

## Data Availability Statement

The datasets generated for this study can be found in the Publicly available datasets were analyzed in this study. This data can be found at: https://www.ncbi.nlm.nih.gov/geo/query/acc.cgi?acc=GSE26886 https://www.ncbi.nlm.nih.gov/geo/query/acc.cgi?acc=GSE1420 https://www.ncbi.nlm.nih.gov/geo/query/acc.cgi?acc=GSE37200 https://www.ncbi.nlm.nih.gov/geo/query/acc.cgi?acc=GSE37201 https://portal.gdc.cancer.gov/exploration?filters=%7B%22op%22%3A%22and%22%2C%22content%22%3A%5B%7B%22op%22%3A%22in%22%2C%22content%22%3A%7B%22field%22%3A%22cases.primary_site%22%2C%22value%22%3A%5B%22esophagus%22%5D%7D%7D%5D%7D https://www.proteinatlas.org/ENSG00000136875-PRPF4/tissue/esophagus.

https://www.proteinatlas.org/ENSG00000136450-SRSF1/tissue/esophagus https://www.proteinatlas.org/ENSG00000099783-HNRNPM/tissue/esophagus https://www.proteinatlas.org/ENSG00000135829-DHX9/tissue/esophagus

https://www.proteinatlas.org/ENSG00000115942-ORC2/tissue/esophagus.

## Author Contributions

DW, GS, and AN conceived and designed the experiment. AN, AK, and SK performed the meta-analysis and network analysis. AN and GS performed the WGCNA. AN, AK, and GS aided in preparation of the figures and supplemental materials. AN and SK performed Cytoscape analysis. AN, SK and GS compiled the manuscript. WC and DW provided expert advice in the study, performed editing and proofreading of the manuscript. All authors contributed to the study and approved the submitted version.

## Funding

The authors are thankful for financial support from the Ministry of Science and Technology of China (Grant No.: 2016YFA0501703), the National Natural Science Foundation of China (Contract No. 61832019, 61503244), the Science and Technology Commission of Shanghai Municipality (Grant: 19430750600), Henan Natural Science (Grant No.:162300410060), as well as SJTU JiRLMDS Joint Research Funds for Medical and Engineering and Scientific Research at Shanghai Jiao Tong University (YG2017ZD14) to DQ.W; Doctoral Study Scholarship from Shanghai Jiao Tong University to ASN; China Postdoctoral Science Foundation (Grant No.: 2018M632766) to GS; Henan Province Postdoctoral Science Foundation (Grant No.: 001802029 & 001803035) to SK and GS.

## Conflict of Interest

The authors declare that the research was conducted in the absence of any commercial or financial relationships that could be construed as a potential conflict of interest.

## Footnotes

1
http://www.ncbi.nlm.nih.gov/geo

2
https://www.ebi.ac.uk/arrayexpress/

3
https://portal.gdc.cancer.gov/

4
https://cytoscape.org/

5
https://string-db.org/

6
http://bioinfo.henu.edu.cn/EAC/EACList.jsp

7
https://www.proteinatlas.org/
