# Supplementary material for: Integrated PPI- and WGCNA-Retrieval of Hub Gene Signatures Shared Between Barrett's Esophagus and Esophageal Adenocarcinoma
Source: Front Pharmacol. 2020 Jul 31;11:881. doi: 10.3389/fphar.2020.00881 (PMC7438937; doi:10.3389/fphar.2020.00881)
Supplement: Supplementary file 1 [file DataSheet_1.pdf]

Supplementary Material

1.1 Supplementary Figures

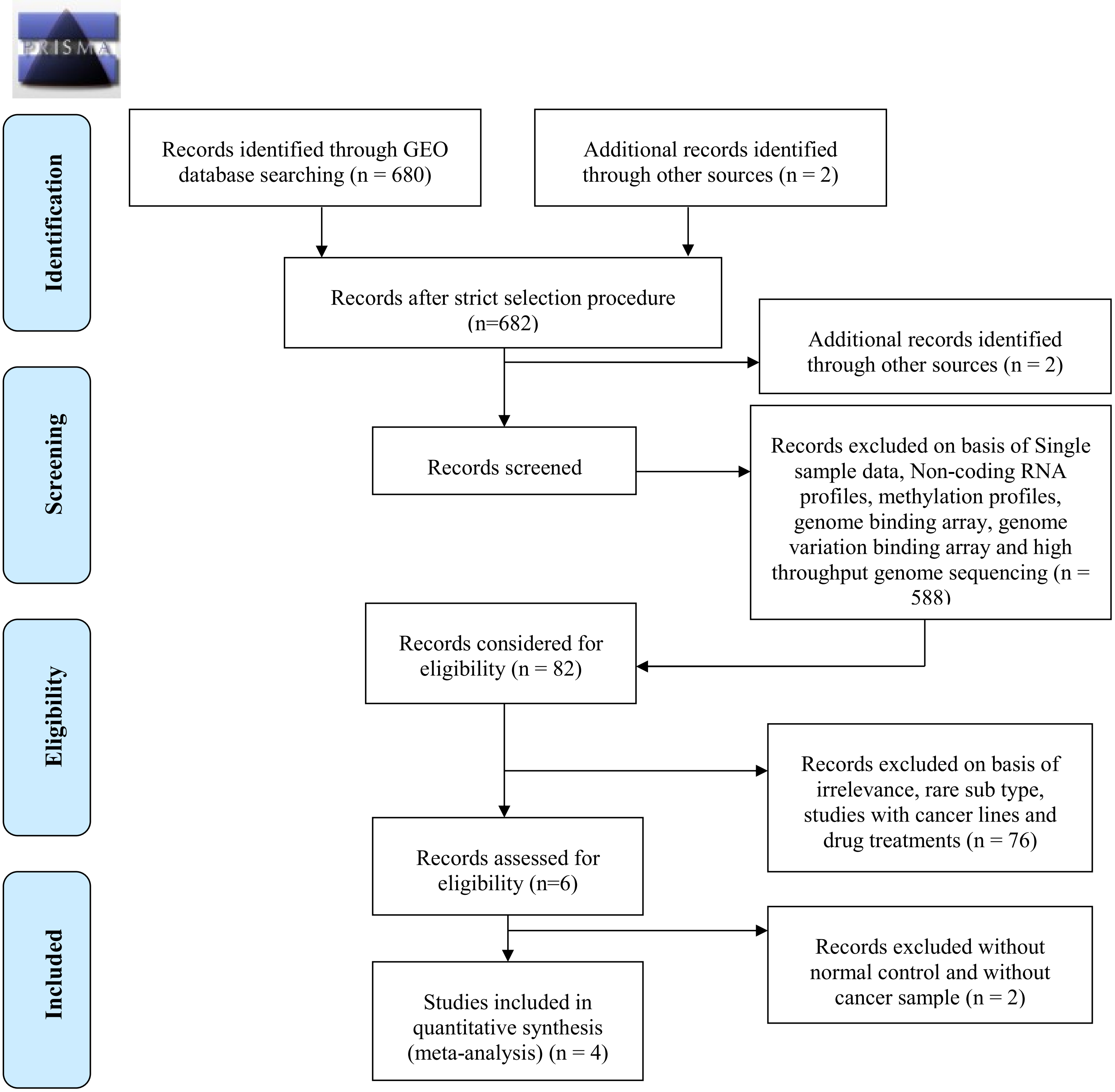

Supplementary Figure 1. PRISMA flow chart of the microarray meta-analysis. BE and EAC related GEO datasets eligible selection.

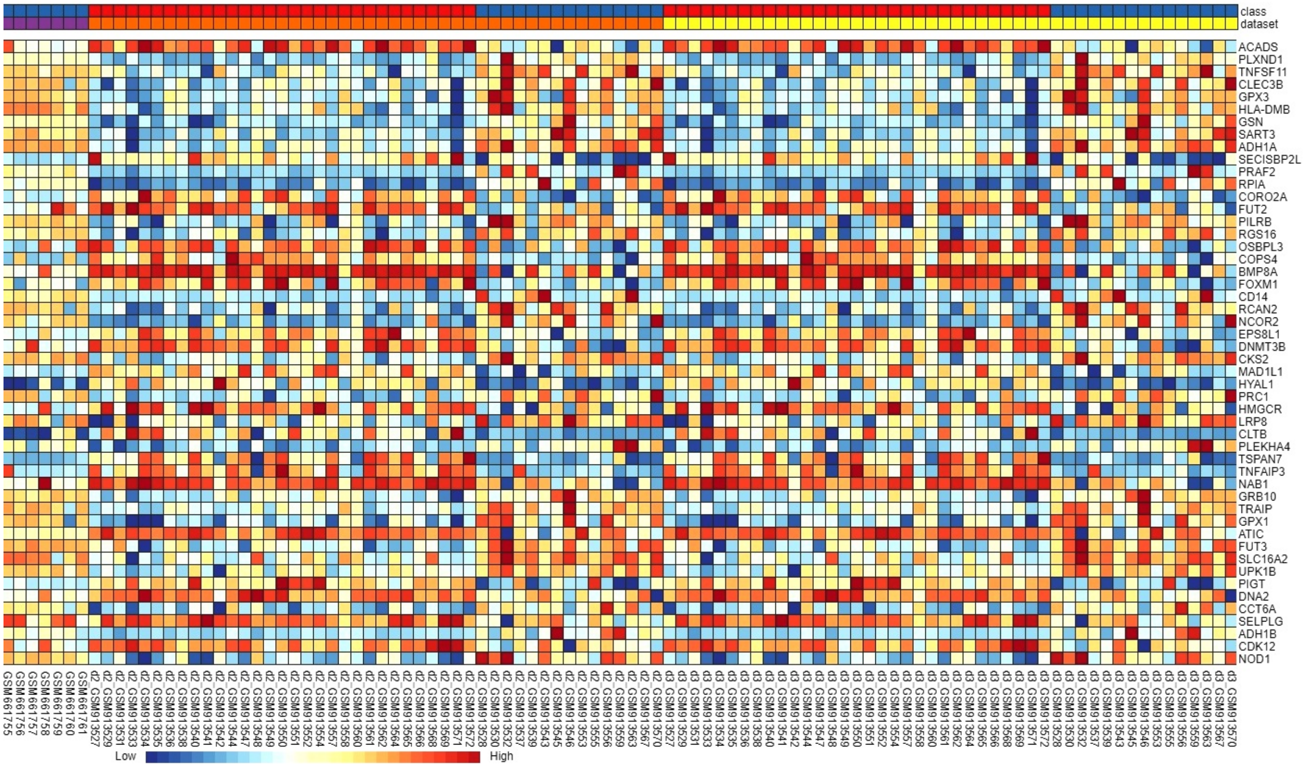

Supplementary Figure 2. Heatmap clustering of DEGs. Major DEGs across all the datasets were represented in red, orange, and yellow in color in a heatmap. Gray indicates that the respective gene is not present in the gene list

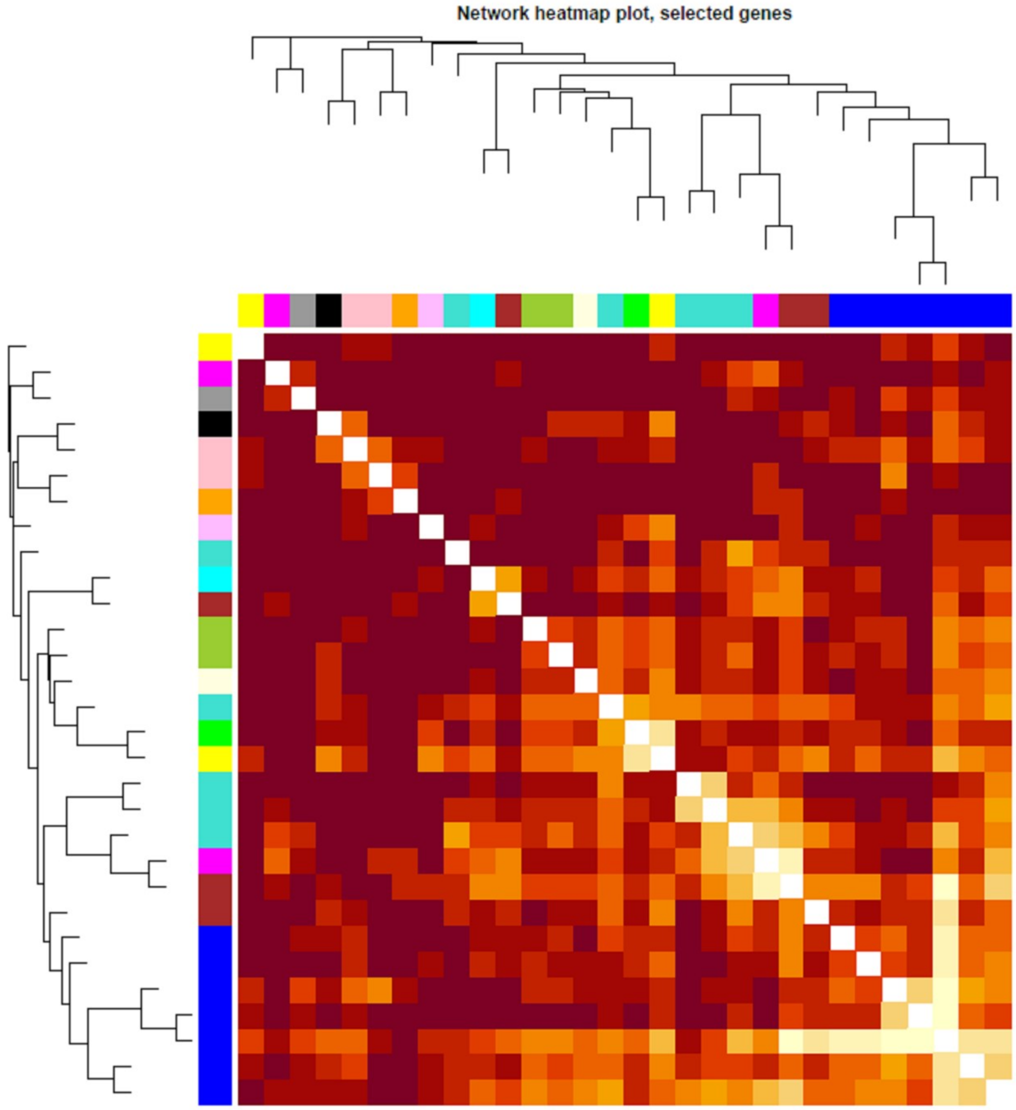

Supplementary Figure 3. Visualizing the gene network using a heatmap plot. The heatmap depicts the Topological Overlap Matrix (TOM) among all genes in the analysis. Light color represents low overlap and the progressively darker red color represents a higher overlap. Blocks of darker colors along the diagonal are the modules. The gene dendrogram and module assignment are also shown long the left side and the top.

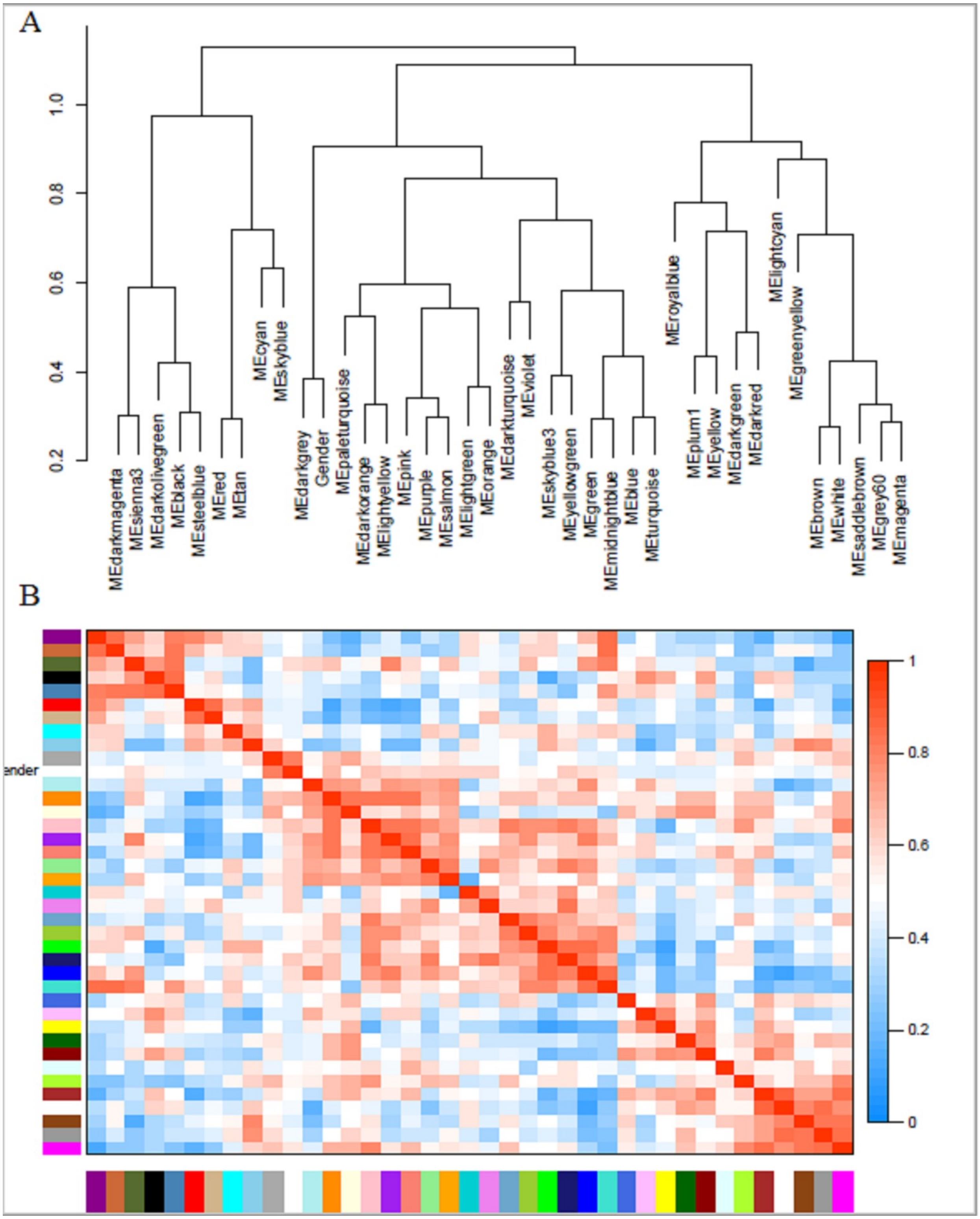

Supplementary Figure 4. Visualization of the eigengene network representing the relationships among the modules and the clinical trait weight. Panel (A) shows a hierarchical clustering dendrogram of the eigengenes in which the dissimilarity of eigengenes E_I, E_J is given by $1 - \text{cor}(E_I ;E_J)$. The heatmap in panel (B) shows the eigengene adjacency $A_{IJ} = (1 + \text{cor}(E_I ;E_J))=2$.

1.2 SupplementaryTables

Supplementary Table 1.WGCNA module genes

Gene ID	Gene Name	WGCNA
51573	glycerophosphodiester phosphodiesterase 1(GDE1)	GDE1
55231	coiled-coil domain containing 87(CCDC87)	CCDC87
79800	calcium responsive transcription factor(CARF)	CARF
4496	metallothionein 1H(MT1H)	MT1H
4493	metallothionein 1E(MT1E)	MT1E
8416	annexin A9(ANXA9)	ANXA9
645745	metallothionein 1H-like 1(MT1HL1)	MT1HL1
4494	metallothionein 1F(MT1F)	MT1F
1314	coatomer protein complex subunit alpha(COPA)	COPA
1416	crystallin beta B2 pseudogene 1(CRYBB2P1)	CRYBB2P1
5375	peripheral myelin protein 2(PMP2)	PMP2
51086	TNNI3 interacting kinase(TNNI3K)	TNNI3K
79088	zinc finger protein 426(ZNF426)	ZNF426
8323	frizzled class receptor 6(FZD6)	FZD6
3485	insulin like growth factor binding protein 2(IGFBP2)	IGFBP2
22800	related RAS viral (r-ras) oncogene homolog 2(RRAS2)	RRAS2
5745	parathyroid hormone 1 receptor(PTH1R)	PTH1R
4213	Meishomeobox 3 pseudogene 1(MEIS3P1)	MEIS3P1
23780	apolipoprotein L2(APOL2)	APOL2
55853	IDI2 antisense RNA 1(IDI2-AS1)	IDI2-AS1
85865	GTP binding protein 10(GTPBP10)	GTPBP10
375035	SFT2 domain containing 2(SFT2D2)	SFT2D2
4884	neuronal pentraxin 1(NPTX1)	NPTX1
4893	neuroblastoma RAS viral oncogene homolog(NRAS)	NRAS
8725	URI1, prefoldin like chaperone(URI1)	URI1
4502	metallothionein 2A(MT2A)	MT2A
2023	enolase 1(ENO1)	ENO1
4501	metallothionein 1X(MT1X)	MT1X
4499	metallothionein 1M(MT1M)	MT1M
29907	sorting nexin 15(SNX15)	SNX15
1457	casein kinase 2 alpha 1(CSNK2A1)	CSNK2A1
10321	cysteine rich secretory protein 3(CRISP3)	CRISP3
23530	nicotinamide nucleotide transhydrogenase(NNT)	NNT
166647	adhesion G protein-coupled receptor A3(ADGRA3)	ADGRA3
51398	WD repeat domain 83 opposite strand(WDR83OS)	WDR83OS
3840	karyopherin subunit alpha 4(KPNA4)	KPNA4
25771	TBC1 domain family member 22A(TBC1D22A)	TBC1D22A
10085	EGF like repeats and discoidin domains 3(EDIL3)	EDIL3
6470	serine hydroxymethyltransferase 1(SHMT1)	SHMT1
54682	MANSC domain containing 1(MANSC1)	MANSC1
51298	theg spermatid protein(THEG)	THEG
9991	polypyrimidine tract binding protein 3(PTBP3)	PTPB3

10313	reticulon 3(RTN3)	RTN3
3839	karyopherin subunit alpha 3(KPNA3)	KPNA3
5245	prohibitin(PHB)	PHB
10492	synaptotagmin binding cytoplasmic RNA interacting protein(SYNCRIP)	SYNCRIP
3835	kinesin family member 22(KIF22)	KIF22
10210	TOP1 binding arginine/serine rich protein(TOPORS)	TOPORS
9889	zinc finger BED-type containing 4(ZBED4)	ZBED4
6382	syndecan 1(SDC1)	SDC1
27291	R3H domain and coiled-coil containing 1 like(R3HCC1L)	R3HCC1L
10598	activator of Hsp90 ATPase activity 1(AHSA1)	AHSA1
2969	general transcription factor IIi(GTF2I)	GTF2I
10243	gephyrin(GPHN)	GPHN
3627	C-X-C motif chemokine ligand 10(CXCL10)	CXCL10
55787	taxilin gamma(TXLNG)	TXLNG
9071	claudin 10(CLDN10)	CLDN10
10850	C-C motif chemokine ligand 27(CCL27)	CCL27
26298	ETS homologous factor(EHF)	EHF
8836	gamma-glutamyl hydrolase(GGH)	GGH
55334	solute carrier family 39 member 9(SLC39A9)	SLC39A9
26030	pleckstrin homology and RhoGEF domain containing G3(PLEKHG3)	PLEKHG3
10057	ATP binding cassette subfamily C member 5(ABCC5)	ABCC5
10963	stress induced phosphoprotein 1(STIP1)	STIP1
55891	lens epithelial protein(LENEP)	LENEP
23366	KIAA0895(KIAA0895)	KIAA0895
56898	3-hydroxybutyrate dehydrogenase, type 2(BDH2)	BDH2
1E+08	TOPORS antisense RNA 1(TOPORS-AS1)	TOPORS-AS1
23682	RAB38, member RAS oncogene family(RAB38)	RAB38
6192	ribosomal protein S4, Y-linked 1(RPS4Y1)	RPS4Y1
3957	galectin 2(LGALS2)	LGALS2
51124	immediate early response 3 interacting protein 1(IER3IP1)	IER3IP1
2561	gamma-aminobutyric acid type A receptor beta2 subunit(GABRB2)	GABRB2
2359	formyl peptide receptor 3(FPR3)	FPR3
51557	lengsin, lens protein with glutamine synthetase domain(LGSN)	LGSN
1607	diacylglycerol kinase beta(DGKB)	DGKB
55258	threonine synthase like 2(THNSL2)	THNSL2
10362	high mobility group 20B(HMG20B)	HMG20B
4734	neural precursor cell expressed, developmentally down-regulated 4, E3 ubiquitin protein ligase(NEDD4)	NEDD4
28959	transmembrane protein 176B(TMEM176B)	TMEM176B
7770	zinc finger protein 227(ZNF227)	ZNF227
246126	taxilin gamma pseudogene, Y-linked(TXLNGY)	TXLNGY
57097	poly(ADP-ribose) polymerase family member 11(PARP11)	PARP11
64963	mitochondrial ribosomal protein S11(MRPS11)	MRPS11
9716	aquarius intron-binding spliceosomal factor(AQR)	AQR
10773	zinc finger and BTB domain containing 6(ZBTB6)	ZBTB6
10775	POP4 homolog, ribonuclease P/MRP subunit(POP4)	POP4
57194	ATPase phospholipid transporting 10A (putative)(ATP10A)	ATP10A

5304	prolactin induced protein(PIP)	PIP
57466	SR-related CTD associated factor 4(SCAF4)	SCAF4
48	aconitase 1(ACO1)	ACO1
11212	proline synthetasecotranscribed homolog (bacterial)(PROSC)	PROSC
4670	heterogeneous nuclear ribonucleoprotein M(HNRNPM)	HNRNPM
3273	histidine rich glycoprotein(HRG)	HRG
11118	butyrophilin subfamily 3 member A2(BTN3A2)	BTN3A2
9087	thymosin beta 4, Y-linked(TMSB4Y)	TMSB4Y
10842	protein phosphatase 1 regulatory subunit 17(PPP1R17)	PPP1R17
7761	zinc finger protein 214(ZNF214)	ZNF214
4999	origin recognition complex subunit 2(ORC2)	ORC2
10640	exocyst complex component 5(EXOC5)	EXOC5
27159	chitinase, acidic(CHIA)	CHIA
7263	thiosulfate sulfurtransferase(TST)	TST
54	acid phosphatase 5, tartrate resistant(ACP5)	ACP5
55635	DEP domain containing 1(DEPDC1)	DEPDC1
7290	histone cell cycle regulator(HIRA)	HIRA
10130	protein disulfide isomerase family A member 6(PDIA6)	PDIA6
51003	mediator complex subunit 31(MED31)	MED31
3149	high mobility group box 3(HMGB3)	HMGB3
3146	high mobility group box 1(HMGB1)	HMGB1
3005	H1 histone family member 0(H1F0)	H1FO
5161	pyruvate dehydrogenase (lipoamide) alpha 2(PDHA2)	PDHA2
5019	3-oxoacid CoA-transferase 1(OXCT1)	OXCT1
9141	programmed cell death 5(PDCD5)	PDCD5
1387	CREB binding protein(CREBBP)	CREBBP
54708	membrane associated ring-CH-type finger 5(MARCH5)	MAR
54810	GIPC PDZ domain containing family member 2(GIPC2)	GIPC2
23302	WSC domain containing 1(WSCD1)	WSCD1
55028	chromosome 17 open reading frame 80(C17orf80)	C17ORF80
4314	matrix metallopeptidase 3(MMP3)	MMP3
4312	matrix metallopeptidase 1(MMP1)	MMP1
4291	myeloid leukemia factor 1(MLF1)	MLF1
1385	cAMP responsive element binding protein 1(CREB1)	CREB1
56851	ER membrane protein complex subunit 7(EMC7)	EMC7
54922	Ras interacting protein 1(RASIP1)	RASIP1
26355	family with sequence similarity 162 member A(FAM162A)	FAM162A
8295	transformation/transcription domain associated protein(TRRAP)	TRRAP
10525	hypoxia up-regulated 1(HYOU1)	HYOU1
26280	interleukin 1 receptor accessory protein like 2(IL1RAPL2)	IL1RAPL2
4023	lipoprotein lipase(LPL)	LPL
2785	G protein subunit gamma 3(GNG3)	GNG3
28999	Kruppel like factor 15(KLF15)	KLF15
9774	BCL2 associated transcription factor 1(BCLAF1)	BCLAF1
10279	protease, serine 16(PRSS16)	PRSS16
3320	heat shock protein 90 alpha family class A member 1(HSP90AA1)	HSP90AA1
4430	myosin IB(MYO1B)	MYO1B
1791	DNA nucleotidylexotransferase(DNTT)	DNTT
4946	ornithine decarboxylase antizyme 1(OAZ1)	OAZ1

60558	GUF1 homolog, GTPase(GUF1)	GUF1
1267	2',3'-cyclic nucleotide 3' phosphodiesterase(CNP)	CNP
23586	DExD/H-box helicase 58(DDX58)	DDX58
23587	elongator acetyltransferase complex subunit 5(ELP5)	ELP5
5151	phosphodiesterase 8A(PDE8A)	PDE8A
9377	cytochrome c oxidase subunit 5A(COX5A)	COX5A
4014	loricrin(LOR)	LOR
26057	ankyrin repeat domain 17(ANKRD17)	ANKRD17
4179	CD46 molecule(CD46)	CD46
22865	SLIT and NTRK like family member 3(SLITRK3)	SLITRK3
5158	phosphodiesterase 6B(PDE6B)	PDE6B
10686	claudin 16(CLDN16)	CLDN16
291	solute carrier family 25 member 4(SLC25A4)	SLC25A4
3329	heat shock protein family D (Hsp60) member 1(HSPD1)	HSPD1
1397	cysteine rich protein 2(CRIP2)	CRIP2
995	cell division cycle 25C(CDC25C)	CDC25C
51319	arginine and serine rich coiled-coil 1(RSRC1)	RSRC1
29766	tropomodulin 3(TMOD3)	TMOD3
3231	homeobox D1(HOXD1)	HOXD1
79184	BRCA1/BRCA2-containing complex subunit 3(BRCC3)	BRCC3
79644	steroid 5 alpha-reductase 3(SRD5A3)	SRD5A3
26511	cysteine rich hydrophobic domain 2(CHIC2)	CHIC2
56672	A-kinase interacting protein 1(AKIP1)	AKIP1
642	bleomycin hydrolase(BLMH)	BLMH
26121	pre-mRNA processing factor 31(PRPF31)	PRPF31
2911	glutamate metabotropic receptor 1(GRM1)	GRM1
23409	sirtuin 4(SIRT4)	SIRT4
80143	suppressor of IKBKE 1(SIKE1)	SIKE1
9100	ubiquitin specific peptidase 10(USP10)	USP10
219595	folate hydrolase 1B(FOLH1B)	FOLH1B
3191	heterogeneous nuclear ribonucleoprotein L(HNRNPL)	HNRNPL
29063	zinc finger CCHC-type containing 4(ZCCHC4)	ZCCHC4
5887	RAD23 homolog B, nucleotide excision repair protein(RAD23B)	RAD23B
5264	phytanoyl-CoA 2-hydroxylase(PHYH)	PHYH
7018	transferrin(TF)	TF
5885	RAD21 cohesin complex component(RAD21)	RAD21
79656	BEN domain containing 5(BEND5)	BEND5
11248	neurexophilin 3(NXPH3)	NXPH3
6732	SRSF protein kinase 1(SRPK1)	SRPK1
26211	olfactory receptor family 2 subfamily F member 1 (gene/pseudogene)(OR2F1)	OR2F1
7503	X inactive specific transcript (non-protein coding)(XIST)	XIST
373	tripartite motif containing 23(TRIM23)	TRIM23
1660	DExH-box helicase 9(DHX9)	DHX9
84900	ring finger protein, transmembrane 2(RNFT2)	RNFT2
9113	large tumor suppressor kinase 1(LATS1)	LATS1
3183	heterogeneous nuclear ribonucleoprotein C (C1/C2)(HNRNPC)	HNRNPC
353189	solute carrier organic anion transporter family member	SLCO4C1

	4C1(SLCO4C1)	
1E+08	psoriasis associated non-protein coding RNA induced by stress(PRINS)	PRINS
5594	mitogen-activated protein kinase 1(MAPK1)	MAPK1
762	carbonic anhydrase 4(CA4)	CA4
1749	distal-less homeobox 5(DLX5)	DLX5
6338	sodium channel epithelial 1 beta subunit(SCNN1B)	SCNN1B
363	aquaporin 6(AQP6)	AQP6
3456	interferon beta 1(IFNB1)	IFNB1
9128	pre-mRNA processing factor 4(PRPF4)	PRPF4
64232	membrane spanning 4-domains A5(MS4A5)	MS4A5
5868	RAB5A, member RAS oncogene family(RAB5A)	RAB54
79931	TNFAIP3 interacting protein 3(TNIP3)	TNIP3
753	low density lipoprotein receptor class A domain containing 4(LDLRAD4)	LDLRAD4
22824	heat shock protein family A (Hsp70) member 4 like(HSPA4L)	HSPA4L
2923	protein disulfide isomerase family A member 3(PDIA3)	PDIA3
4712	NADH:ubiquinone oxidoreductase subunit B6(NDUFB6)	NDUFB6
2922	gastrin releasing peptide(GRP)	GRP
1964	eukaryotic translation initiation factor 1A, X-linked(EIF1AX)	EIF1AX
6857	synaptotagmin 1(SYT1)	SYT1
9667	scaffold attachment factor B2(SAFB2)	SAFB2
51350	keratin 76(KRT76)	KRT76
1677	DNA fragmentation factor subunit beta(DFFB)	DFFB
91752	zinc finger protein 804A(ZNF804A)	ZNF804A
54842	major facilitator superfamily domain containing 6(MFSD6)	MSFD6
6426	serine and arginine rich splicing factor 1(SRSF1)	SRSF1
9128	pre-mRNA processing factor 4(PRPF4)	PRPF4
64232	membrane spanning 4-domains A5(MS4A5)	MS4A5
5868	RAB5A, member RAS oncogene family(RAB5A)	RAB54
79931	TNFAIP3 interacting protein 3(TNIP3)	TNIP3
753	low density lipoprotein receptor class A domain containing 4(LDLRAD4)	LDLRAD4
22824	heat shock protein family A (Hsp70) member 4 like(HSPA4L)	HSPA4L
2923	protein disulfide isomerase family A member 3(PDIA3)	PDIA3
4712	NADH:ubiquinone oxidoreductase subunit B6(NDUFB6)	NDUFB6
2922	gastrin releasing peptide(GRP)	GRP
1964	eukaryotic translation initiation factor 1A, X-linked(EIF1AX)	EIF1AX
6857	synaptotagmin 1(SYT1)	SYT1
9667	scaffold attachment factor B2(SAFB2)	SAFB2
51350	keratin 76(KRT76)	KRT76
1677	DNA fragmentation factor subunit beta(DFFB)	DFFB
91752	zinc finger protein 804A(ZNF804A)	ZNF804A
54842	major facilitator superfamily domain containing 6(MFSD6)	MSFD6
6426	serine and arginine rich splicing factor 1(SRSF1)	SRSF1
